# ADLAuth: Passive Authentication Based on Activity of Daily Living Using Heterogeneous Sensing in Smart Cities

**DOI:** 10.3390/s19112466

**Published:** 2019-05-29

**Authors:** Maryam Naseer Malik, Muhammad Awais Azam, Muhammad Ehatisham-Ul-Haq, Waleed Ejaz, Asra Khalid

**Affiliations:** 1 Faculty of Telecom and Information Engineering, University of Engineering and Technology, Taxila, Punjab 47050, Pakistan; maryamnaseer01@gmail.com (M.N.M.); awais.azam@uettaxila.edu.pk (M.A.A.); ehatishamuet@gmail.com (M.E.-U.-H.); 2 Department of Applied Science & Engineering, Thompson Rivers University, Kamloops, BC V2C 0C8, Canada; 3 Department of Computer Science, COMSATS University, Wah Cantt. 47040, Pakistan; asra.khalid@ciitwah.edu.pk

**Keywords:** smartphone, continuous user authentication, behavioral biometrics, physical activity, user identification, heterogeneous sensors, ubiquitous computing, smart cities

## Abstract

The Internet of Things is a rapidly growing paradigm for smart cities that provides a way of communication, identification, and sensing capabilities among physically distributed devices. With the evolution of the Internet of Things (IoTs), user dependence on smart systems and services, such as smart appliances, smartphone, security, and healthcare applications, has been increased. This demands secure authentication mechanisms to preserve the users’ privacy when interacting with smart devices. This paper proposes a heterogeneous framework “ADLAuth” for passive and implicit authentication of the user using either a smartphone’s built-in sensor or wearable sensors by analyzing the physical activity patterns of the users. Multiclass machine learning algorithms are applied to users’ identity verification. Analyses are performed on three different datasets of heterogeneous sensors for a diverse number of activities. A series of experiments have been performed to test the effectiveness of the proposed framework. The results demonstrate the better performance of the proposed scheme compared to existing work for user authentication.

## 1. Introduction

The Internet of Things (IoT) is a network that provides a notion of ubiquitously connecting smart sensors, smart devices, and other daily living physical objects, thus giving rise to smart cities. Unlike the traditional internet that offers a connection between the people and a network, IoT aims to provide the connectivity among devices, objects, and human beings for enabling diverse applications for smart cities such as user identification, monitoring, tracking, and control. With flourishing advancement in the field of IoT, an emerging paradigm consisting of ubiquitous sensing has been introduced. The rapid increase in smart sensing capabilities has led to an outburst in the use of smart systems and services, which has encouraged researchers and developers to think about the development of smart cities. As a result, researchers are focused on presenting novel ideas for smart services and smart devices, which can extract information from IoT generated data for automation and better decision making. Nowadays, most of the people during their daily routine rely on smart systems and services in terms of social contact, environment, healthcare, transportation, and entertainment. Smart cities as a dominant area of IoT must be able to provide high-quality smart services to the people in terms of social contact, environment, healthcare, transportation, and entertainment. Smart buildings are one of the core blocks of smart cities as people nowadays spending most of the time in indoor activities for shopping, education, and work. Therefore, smart services should facilitate and meet the needs of the inhabitants of smart cities. At one end, the development of smart systems and services, such as smart appliances, smart home [1], security and surveillance applications, healthcare and monitoring such as telecare medicine information system [2] is providing easiness in the life of people. On the other hand, there are privacy issues arising due to these systems as they contain some sensitive and vital user information. Similarly, besides their traditional voice communication, smartphones are providing a common platform for numerous online services such as email, online transactions, and e-commerce. Consequently, a large amount of personal information, such as contact details, call logs, photos, and bank account number, are being generated and stored on smartphones. Hence, potential threats to the owners of the accounts have increased significantly. Also, smartphones are quite susceptible to probably being lost, stolen, or easily accessed by non-owners. As much as these devices provide substantial benefits for users in everyday life, their privacy continues to be a major concern. Hence, it has now become essential to explore authentication mechanisms that can maintain the privacy of sensitive information, which is made available through these devices.

Authentication is the process of identifying the real user of the system [3]. User authentication can be broadly classified into three categories: (i) knowledge-based; “what user knows” (involves something that user must know like password, Personal Identification Numbers (PINs), an ID number, or answer to a security question etc.), (ii) object-based; “what user has” (which involves something that user possesses such as ID card, token, keys, badges, etc.), (iii) biometric; “who user is” (which denotes the behavioral characteristics that can be represented by one or more physical or behavioral attributes, e.g., fingerprint, face, iris, keystroke dynamics, etc.) [4]. Passwords and PIN codes are the conventional methods used for user authentication, which are explicit mechanisms and require the active participation of the user. Simple passwords can be easily remembered; however, they can be guessed too as there is a risk of information disclosure (if the password is some useful information related to the user) also. Hence, passwords are weak and vulnerable to guessing attacks [5,6].

Complex passwords can also be obtained by determining the screen taps location using accelerometer and gyroscope sensor readings [7]. Long secure passwords are generally more annoying to the users because of the effort required for their input. PINs are easier to remember than passwords. However, they are less secure and can be guessed more quickly [8,9]. It is estimated that an average user takes almost 4.7 s to unlock his/her smartphone through a PIN [10]. Another most common method used in smartphones for authentication is the pattern lock, which provides an easy way to login to the device. Unfortunately, they are vulnerable to side-channel attacks and can be guessed by determining the trace of the fingerprints on the screen after the pattern has been drawn. Biometric authentication has also been employed for smart devices and smart systems, which can be divided into two categories: physiological and behavioral. Commonly used authentication methods involving physiological biometric include face lock, iris recognition, and fingerprint scan. The main drawback of using physiological biometrics for authentication purpose is that these physiological characteristics can be duplicated and altered. For instance, hand geometry and fingerprints can be recreated in plastic [11]. Scars and bruises can change the fingerprints, and different poses of the face can create confusion in the face recognition system [12]. Moreover, physiological biometric methods using fingerprint and iris recognition require the support of some additional hardware for input. In addition, these methods are active authentication methods and require the user to frequently and actively participate in the system for getting access to any smart system, which becomes annoying and tedious to the users. As a result, users prefer to use fewer privacy impediments. To overcome these challenges relating to user authentication, the implementation of continuous authentication based on behavioral biometrics offers a way for passive user authentication.

Passive authentication does not necessitate the direct involvement of the user in the system. Instead, it is concerned with the behavioral traits of the user and continually monitors the user and identify user’s authenticity rather than authenticating the user once on the entry point, hence enhancing system security. With the latest advancement in IoTs, sensor-based biometric authentication mechanisms are proposed in which wearable sensors are attached to the user’s body for continuously monitoring the user. On-body wearable sensors have the advantage of mobility and are always with the user as opposed to traditional biometric systems, which are fixed at a location. Sensor-based wearable devices come in different forms focusing on accessories and clothing that people use in daily routines. These accessories and clothing comprise wrist-worn bands, smart jewelry, straps, head-mounted devices, E-Textiles, and E-Patches [13]. Wearable sensors are attached to the body unobtrusively, which continuously authenticate the user based on the behavioral and physiological signals captured from the user’s body. Smartphone-based behavioral biometrics also grasped momentous attention in recent years. User authentication scheme based on hand-writing pattern [14,15], touch behavior [16], gait patterns [17,18], and GPS location patterns [19] are proposed, which require minimal user interaction with no additional hardware support. With the latest advancement in mobile sensing technology, behavioral-based authentication mechanisms using the motion sensors, embedded in the smartphone, have become quite popular. The behavioral patterns of the user are monitored throughout the phone usage process, even when the phone is in the locked condition, which improves the privacy of the user. In sensor-based authentication, the experimental context is complex in terms of the diverse range of sensors, actions performed by the user, and the position of the device used in data collection [20]. A few research studies on smartphone user authentication focused on the recognition of activities of daily living (ADL) for authenticating smartphone users and analyzed the effect of smartphone placement on the recognition performance [20,21,22,23]. Behavioral biometrics like gait [18,24] and touch behavior [16] have also been used for smartphone user authentication. Although these daily living activities are prevalent in real life, however, the authentication mechanism cannot be limited to only those activities. For example, due to any physical and mental health condition, the way of performing these activities can be changed. The walking pattern of the user can vary due to changing footwear; in this case, the user cannot be identified using simple walking. Similarly, sitting and standing activities have a static nature, and it might be possible that they give low accuracy in identification. In this case, a postural transition like sit-to-stand and stand-to-sit can assist in authenticating the user. Also, the probability of occurrence is different for different activities in real life, so more activities need to be incorporated for viable authentication.

In this paper, a heterogeneous sensing framework ADLAuth (ADL-based authentication) is proposed to provide passive and continuous user authentication by analyzing and recognizing the unique characteristics of the physical activity patterns of a user. For this purpose, different ADL and their changing patterns are considered in this study for providing user authentication. The data from smartphone motion sensors (accelerometer, gyroscope, and orientation sensors) and wearable inertial sensors are used to validate the users on the basis of ADL. The ADLAuth framework evaluated three different datasets for physical activity recognition of users for their identification. These datasets were MobiAct [25], Human Activity Recognition using Smartphone dataset (HAR) [26], and (Physical Activity Monitoring Dataset) PAMAP2 [27], containing different types of daily life activities performed by a number of users. After data preprocessing, a set of time domain and frequency domain features are extracted for user recognition based on ADL patterns. Three supervised machine learning classifiers, support vector machine (SVM), decision tree (DT), and random forest (RF) were used to classify the extracted features into different users. The results of the ADLAuth framework showed that the proposed method is feasible and reliable for passive user authentication. The main contribution of our work is as follows:

- A novel framework ADLAuth is proposed for passive and continuous user authentication, which is based on the recognition of daily life activity patterns.
- Varying patterns of different activities are incorporated into the proposed scheme for assisting in-the-wild user authentication.
- To demonstrate the independence of our method on the type of sensors (either smartphone sensors or wearable inertial sensors), a comprehensive evaluation is provided on three datasets that include data from smartphone sensors as well as wearable sensors of different activities.
- An analysis of different time domain and frequency domain features is provided so that more implicit information is exploited using the users’ ADL.

The rest of the paper is organized as follows: Section 2 describes the related work done for activity recognition and authentication of users using built-in smartphone sensors and wearable sensors. Section 3 describes in detail the datasets used and proposed framework that is evaluated on those datasets. Section 4 depicts the analysis of the performance of the proposed framework. Section 5 gives the concluding remarks and directions for future research.

## 2. Related Work

The aim of activity recognition is to describe how human beings perform their physical activity by analyzing the data collected through multiple sensors. Human activity recognition has many important applications that do not only monitor human activities but also help to understand those activities (e.g., surveillance environments) better. With the development in computing and sensing technology, sensors-based methods for activity recognition have become quite popular as they are cheap and robust in various situations. Multiple on-body sensors have been used at different body positions to recognize human activities [28,29]. In References [30,31], the accelerometer has been used for physical activity recognition by applying different feature extraction algorithms. The accelerometer sensor is used either in its triaxial arrangement or combined with other sensors like a gyroscope, magnetometer, heart rate, or temperature sensors [32]. In Reference [33], the authors recognized human activities using heterogenous sensors at different body positions, i.e., the right pocket, wrist, belt, and arm. Gafurov et al. [34] used a device that was attached to the user’s lower leg and obtained the acceleration signal in three dimensions for authentication. The results were evaluated by implementing histogram similarity and equal cycle length, which achieved an equal error rate (EER) of 5% and 9% respectively. Blasco et al. [35] designed a biometric system using wearable sensors that have potential differences from traditional biometric systems. They provided a feasible solution to biometric verification using wearable sensors composed of acceleration, photoplethysmogram (PPG), electrocardiogram (ECG), and galvanic skin response (GSR). Their result showed an EER of 2% with one-minute of training data using the configuration of ECG, PPG, and GSR. Zhang et al. [36] proposed a parallel ECG-based authentication (PEA) method incorporating hybrid ECG features to improve the authentication accuracy for smart healthcare systems. The experimental results verified that the performance of PEA is comparable to existing approaches. The authors in Reference [37] used wireless body area networks (WBANs) to collect and exchange valuable information regarding physical conditions of the patient. For behavioral authentication of the user, the wearable sensors attached with wrist-worn devices and straps have also been used. In this aspect, an authentication scheme [38] has been carried out on fifteen volunteers, in which a wrist-worn device, i.e., smartwatch, was used to authenticate a user based on his/her gait pattern by examining the accelerometer data. The proposed scheme distinguished an authorized user form an imposter with the authentication accuracy as high as 99.6% and an equal error rate (EER) as low as 2.9%. In Reference [39], the authors utilized a smartwatch to authenticate a user based on the gait pattern. The complete system was implemented on a Samsung Gear Live smartwatch by adopting a new method of sparse fusion to improve accuracy. Yang et al. [40] presented a motion-based authentication scheme using wrist-worn devices with inertial measurement units (IMUs). An android smart watch was used to implement the system. However, these on-body wearable sensors were uncomfortable to wear while performing different activities. Also, they created problems in their adjustment on the user’s body. As a result, smartphone sensors are often used for human activity recognition.

Nowadays, smartphones are equipped with multiple sensors (e.g., accelerometer, gyroscope, orientation, and proximity sensor, etc.), which makes these sensors easily accessible. Because of these advantages, smartphone sensor-based activity recognition has gained a lot of attention in recent years. Researchers have used these three-dimensional sensors for static as well as continuous authentication [20,21,41]. Chen et al. presented a systematic performance analysis of sensor behavior for recognizing human activities using smartphones. Authors developed and implemented both a personalized and generalized model for activity recognition. Extensive results demonstrated that everyone has its own distinct movement patterns. They achieved an *F*-score of 95.95% and 96.26% for the personalized model and generalized model, respectively [23]. In Reference [42], a biometric authentication scheme was proposed, where a user was identified by examining the movement he performs while answering a phone call. The user’s movement was detected by recording accelerometer and orientation sensors data. Muaaz et al. [43] proposed a smartphone-based authentication scheme by examining the user’s gait behavior and observed how the device security was compromised when imitation attacks were performed [43]. The highlight of their work was the collection of malicious data against zero-effort attack in a realistic scenario. In addition to this, Abate et al. [44] investigated carefully about the fusion method. They examined how the fusion of multiple smartphone sensors affects authentication. Their method involved the inspection of sensors like gyroscope sensor, camera, and accelerometer. They conducted their experiment by fusing accelerometer and gyroscope sensors and concluded that only using accelerometer alone gives better authentication results instead of fusing both, which leads to inaccurate results.

Some research works have investigated whether unobtrusive identification or authentication means subjects do not need to perform any particular actions. Kwapisz et al. [45] used the mobile phone-based accelerometer for unobtrusive biometric authorization or identification. Authors used Neural Network and their own strategy of Straw-Man as the learning algorithms and achieved 100% negative and positive authentications of all the five users. Wei-Han Lee and Ruby B. Lee [46] put forward that they achieved high accuracy (90%, up to 95%) by a multi-sensor authentication method with the orientation sensor, accelerometer, and magnetometer. These studies revealed that the fusion of multiple sensors provided better classification results. Additionally, authentication mechanisms must take into consideration more complex contexts. Primo et al. [47] proposed a gait-based authentication scheme and investigated how a different phone position can affect authentication.

In this study, the challenges associated with the existing research to authenticate the user of a smart system through behavioral patterns were analyzed, and to overcome those challenges, we presented ADLAuth framework that utilized data of wearable sensors as well as smartphone sensors to provide an appropriate solution for continuous authentication of the user.

## 3. Methodology

**Figure 1:**
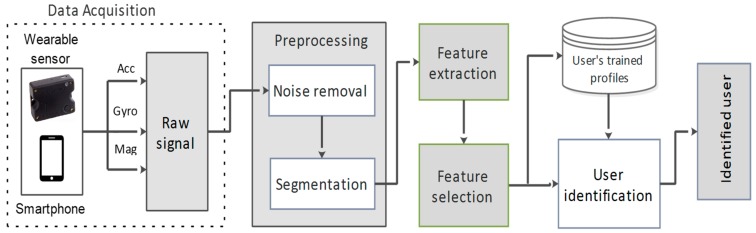
The proposed methodology for user identification.

This section focuses on the procedure adopted to learn and identify the user of a smart system through smartphone built-in and wearable sensors, based on their physical activity patterns. The proposed methodology for ADLAuth framework is shown in Figure 1, which consists of the following five steps: data collection, data preprocessing, feature extraction, feature selection, and user identification. The detail of each section is elaborated below.

### 3.1. Dataset

This section focuses on the datasets used to evaluate this study. Three datasets MobiAct, HAR (Human Activity Recognition using Smartphone dataset), and PAMAP2 (Physical Activity Monitoring Dataset) [27] were used for the evaluation purpose in which two datasets MobiAct and HAR contained the inertial data of smartphone sensors accelerometer, gyroscope, and magnetometer and PAMAP2 dataset was based on wearable sensors at three different body positions. The data was collected from a group of subjects while performing daily routine activities. Table 1 defines the characteristics of these three datasets with only the selected activities used in this study. The selected activities can be categorized into different groups, i.e., static, dynamic, and transitional. The reason for selecting these activities was that authentication can be performed in a better way while incorporating these activities as they are more common in the daily life of a person.

#### 3.1.1. MobiAct Dataset

The MobiAct dataset consists of twelve different activities and four different types of falls. A total of 59 participants performed ADLs. Falls were not considered in this research, and out of eleven ADLs, we only used nine activities that were more related to the authentication purpose. The chosen activities fall in the categories of dynamic, static, and transitional activities. The duration of activities during data collection varied from each other. Three triaxial accelerometers, gyroscope, and orientation sensors were used for data collection. Table 1 defines the complete characteristics of the dataset. The dataset is publicly available and can be obtained from Reference [48].

#### 3.1.2. HAR: Human Activity Recognition Using Smartphone Dataset

HAR is a smartphone-based human activity recognition dataset and is publicly available [26]. A group of 30 participants had performed the experiment of six basic human daily life activities by attaching a smartphone to belt carrying at their waist for the collection of the dataset. The dataset contained the triaxial signals from the device’s embedded sensors of accelerometer and gyroscope at a rate of 50 Hz. The start and end labels were defined for each activity. Duration of all activities were 15 s except walking upstairs and walking downstairs activities, which were of 12 s.

#### 3.1.3. PAMAP2: Physical Activity Monitoring Dataset

The PAMAP2 is a publicly available dataset [49] used for academic research and was initially presented by Reiss and Stricker [27]. This dataset is based on on-body wearable sensors and collected using four sensors, three inertial measurement units (IMUs), and one heart rate monitoring sensor. A total of nine subjects (eight males and one female) participated in the experiment following a data collection protocol. Inertial measuring units were composed of two accelerometers of different scales (±6 g and ±16 g), one gyroscope and one magnetometer at a sampling rate of 100 Hz. Sensors were positioned on the chest, the wrist of the dominant arm and ankle. Eighteen daily living activities were performed, from which only eight activities were used in our work. The nature of the selected activities was categorized as static and dynamic activities.

### 3.2. Data Pre-Processing

Data Denoising and Segmentation

The raw data collected from sensors contained system measurement noise or other unexpected noise owing to the vivacious movements of the user during the experiments. Noisy signal corrupts the useful information contained in the signal. Hence, it was vital to reduce the effect of noise so that meaningful information can be extracted from the signal for further processing. The most common methods used for the filtering includes mean filter, low-pass filter, Wavelet filter, and Gaussian filter [50,51]. In our study, we employed an average smoothing filter for signal denoising by applying the filters along all three dimensions of the accelerometer, gyroscope, and orientation sensors.

After noise removal, the filtered data was segmented into small chunks for further processing using a fixed size sliding window. The filtered continuous signal is divided into equal size segments of 10 s in time with 500 samples at the rate of 50 Hz and 1000 samples at the rate of 100 Hz. Those activities which have a length shorter than 10 s were kept as it was.

### 3.3. Feature Extraction

Feature extraction is an essential step that extracts the vital information from the preprocessed data that can be helpful in recognizing different types of activities and identify differences among users based on activity patterns. “How can the features be extracted?” and “which features to choose?” are important questions and will affect the performance significantly. In this section, after conducting an empirical analysis of the features, a set of 18 features was selected from the time and frequency domain. From the set of fourteen features, autoregression was a feature which returns four coefficients, e.g., 14 features + 4 autoregression coefficients = 18, so the size of the feature vector varied with the dataset. The MobiAct and PAMAP2 datasets contained data of three sensors (acceleration, gyroscope, magnetometer) and features were computed for all three dimensions. The features defined in Equations (11)–(13) gave a single value over all the dimensions of a single sensor’s data. In this case, the size of the feature vector was (18 × 3 + 3) × 3 = 171. Similarly, for the HAR dataset, the size of the feature vector was reduced to (18 × 3 + 3) × 2 = 114 as this dataset contained only two sensors (acceleration and gyroscope). The adopted features for this study were mentioned below with a brief description.

#### 3.3.1. Time Domain Features

- Arithmetic mean represents the mean value of the component contained in the signal.
  (1) $$\bar{s}=\;\frac{1}{N}\sum_{i=1}^{N}s_{i}$$
  where $s_{i}$ represents the *i*th sample value of the original signal and *N* is the total number of samples.
- Minimum amplitude is denoted by $s_{min}$ and shows the minimum value of the signal,
  (2) $$s_{min}=\;min\left(s_{i}\right)$$
- Maximum amplitude is denoted by $s_{max}$ and shows the maximum value of the signal,
  (3) $$s_{max}=\;max\left(s_{i}\right)$$
- Standard deviation, denoted by *std*, depicts how much there is a spread from the mean or expected value of the data. For a low value of standard deviation means that numbers are close to the mean value. *Std* can show the intensity of activities the user performs,
  (4) $$std\left(s\right)=\;\sigma =\sqrt{\frac{1}{N}\sum_{i=1}^{N}{\left(s-\bar{s}\right)}^{2}}$$
- Kurtosis measures tailedness of the probability distribution of real-valued random variables. The standard measure of the kurtosis is based on the fourth moment about the mean where the number of the moment is related to the tail of the distribution of data.
  (5) $$kurtosis\left(s\right)=\overset{N}{\underset{i}{\sum }}\frac{{\left(s_{i}-\bar{s}\right)}^{4}}{N\sigma^{4}}$$
  where σ is the standard deviation.
- Skewness measures the symmetry of the variation of a signal about its mean,
  (6) $$skewness\left(s\right)=\;\overset{N}{\underset{i}{\sum }}\frac{{\left(s_{i}-\bar{s}\right)}^{3}}{N\sigma^{3}}$$
- Signal magnitude area, denoted by *sma*, calculates the magnitude of the triaxial signal.
  (7) $$sma\left(s\right)=\;\frac{1}{3}\sum_{i=1}^{3}\sum_{j=1}^{N}\left|s_{i,j}\right|$$
  where *i* represents the dimension of the triaxial signal and *j* represents the sample value of a particular dimension.
- Median absolute deviation, represented by *mad*, is a robust statistic measure used to show the variability of the data.
  (8) $$mad\left(s\right)=\;median_{i}\left(\left|s_{i}-median_{j\left(s_{j}\right)}\right|\right)$$
  where $median_{j\left(s_{j}\right)}\;$ represents the median value of the signal.
- Interquartile range: In descriptive statistics, the interquartile range is the measure of the variability of data by dividing data into quartiles. It can be determined by calculating the difference between the first and third quartile.
  (9) $$iqr\left(s\right)=\;Q3\left(s\right)-Q1\left(s\right)$$
  where $Q3\left(s\right)$ and $Q1\left(s\right)$ represent the third and first quartile of the signal.
- Autoregression (AR) coefficients are a popular feature of extraction methods. AR modeling attempts to models the signal by using the previous time steps of the signal. AR coefficients give much more detailed information about the signal than mean and variance alone. The given function is used to find the AR coefficients to the 4th order using Burg’s method [52],
  (10) $$a=arburg\left(s,4\right),\;a\epsilon {\mathbb{R}}^{4}$$
- Sum vector magnitude,
  (11) $$\left|s\right|\;=\;\sqrt{s_{x}^{2}+s_{y}^{2}+s_{z}^{2}}$$
- Angle between the *z*-axis and vertical: Calculates the angle between the *z*-axis of the sensor’s data and vertical plane. It results in feature size of R × 1 dimension.
  (12) $$\theta 1=atan2\left(\sqrt{s_{x}^{2}+s_{y}^{2}},s_{z}\right)$$
  where R is the total number of samples over all dimensions in a chunk of sensor’s data that is being segmented.
- Orientation of a person’s trunk,
  (13) $$\theta 2=atan\left(\frac{\sqrt{s_{x}^{2}+s_{y}^{2}}}{s_{z}}\right)$$
- Angle between device and ground,
  (14) $$\theta 3=\sin\left(a\right)$$

#### 3.3.2. Frequency Domain Features

- Max frequency index represented by *maxFreqInd* is the most significant frequency component of the signal,
  (15) $$axFreqInd\left(S\right)=\;arg\;max_{i}\left(S_{i}\right)$$
- Mean frequency: gives the average frequency, calculated as the sum of products of the power spectrum of the signal and divided by the total sum of spectral intensity,
  (16) $$meanfreq\left(S\right)=\;\sum_{i=1}^{N}\left(iS_{i}\right)/\sum_{j=1}^{N}S_{j}$$
- Energy: shows the strength of the signal. At first, the signal is transmitted to the frequency domain then energy was calculated using the formula below [41],
  (17) $$E_{f}=\sum {\left|S\left(f\right)\right|}^{2}$$
- Entropy: shows how much useful information is associated with the signal [41],
  (18) $$H\left(S\left(f\right)\right)=\;-\sum_{i=1}^{N}p_{i}\left(S\left(f\right)\right)\;{log}_{2}p_{i}\left(S\left(f\right)\right)$$
  where $p_{i}$ is the probability of the signal.

### 3.4. Feature Selection

After extracting the specified features above, a feature selection method, known as “Wrapper Subset Evaluation” (WrapperSubsetEval) [53] was applied to the extracted set of features for eliminating redundant features that do not play any vital role in the classification step ahead. The WrapperSubsetEval feature selection method used a learning scheme to evaluate different attributes or set of attributes. A cross-validation scheme was used to approximate the accuracy of the learning classifiers for a different set of features. In our case, we used RF classifier to attribute evaluation with a number of folds equal to 5.

### 3.5. User Identification

After feature extraction, the next step was to choose the suitable classification algorithm to classify the user performing different activities. Classification algorithm plays a vital role in the recognition system, and the right choice of classifiers has a high impact on recognition accuracy. Since every user had different activity patterns and thus the features extracted on those activity patterns gave distinct information about each user. Classifiers learn meaningful information from the extracted features and make decisions based on those features. Three widespread used classifiers: support vector machine (SVM), decision tree (DT), and random forest (RF) were used in this study. Ten-fold cross-validation was used for training and testing of the classifier and the activities performed by the user were classified into different activities groups. Each user had a different activity pattern, so everyone was identified based on the way they perform activities. The main reason for selecting these classifiers was their efficient performance in existing work [20,21,41] for user identification. The detail of each machine learning classifier is described in the following sections.

#### 3.5.1. Support Vector Machine

The support vector machine [54] is a supervised machine learning algorithm used for both regression and classification analysis. SVM classifies data using a hyperplane in one of two categories. In the training phase SVM mapped the data which belonged to each category into high dimensional space by creating a hyperplane which maximizes the distance between two categories. The new data samples were then mapped to the same space based upon the which side of the hyperplane the new sample fell. SVM could also perform non-linear classification efficiently through the kernel trick, i.e., mapping input space into high dimensional feature space.

#### 3.5.2. Decision Tree

The decision tree [55] is a supervised machine learning approach used for regression as well as for classification. The motivation of the decision tree was to build a training model which was used to predict a target variable by learning simple decision rules. The decision rules were constructed from the extracted features of the input data. The understanding of the decision tree algorithm was quite simple compared to another machine learning algorithm, yet highly interpretable. The problem with the decision tree was that it is expensive to add a new sample to the already trained model [56].

#### 3.5.3. Random Forest

Random Forest [57] is an easy to use supervised machine learning algorithm. Due to its simplicity and flexibility, it is a widely used algorithm for classification and regression problems. As its name implies, it created multiple decision trees and added randomness to the model to achieve more stable and accurate results. Another advantage of random forests was that instead of using important features, it selected the best features among the set of features which made the model better. Random forests also avoided the problem of overfitting, which was an issue in deep decision trees. The main limitation with the random forests algorithm was that a large number of trees made it slow for real-time applications.

## 4. Results

This presents the experimental results obtained for the proposed authentication framework. As the proposed scheme focused on heterogeneous sensing for user authentication, hence three different datasets including HAR, MobiAct, and PAMAP2 datasets, were evaluated for the proposed scheme. To perform the continuous authentication for the users, the proposed framework allowed the performance of the recognition of physical activities from both wearables as well as smartphone sensors by analyzing different activities performed by the users, and based on their activity patterns, each user was identified. The performance of the proposed framework was evaluated using three different classifiers: SVM, RF, and DT. The hyperparameters for these classifiers were tuned as follows: A polynomial kernel with sequential minimal optimization (SMO) algorithm [58] and pairwise classification (i.e., 1-vs.-1) approach was used for the SVM classifier. For the RF classifier, a random tree was used with a based leaner, and the number of iterations was set as 100. A pruned C4.5 tree (J48) [59] was used for DT classifier with a number of folds equal to 3.

The selected classifiers were trained for each dataset containing different physical activities and then tested on those activities. Initially, the activities were recognized as performed by different users, and user labels were assigned to each activity. For training and testing purpose, *k*-fold cross-validation was used with *k* = 10. A wrapper feature selection method, i.e., WrapperSubsetEval, was also applied to the extracted set of features, which used the RF learner to assess different subsets of attributes. The hyperparameters were settled for each classifier before the training process. The proposed heterogenous framework was used to exploit three different datasets for user recognition, based on the labeled activity data. The performance of the framework was evaluated in terms of performance metrics F-measure, average accuracy, and Root Mean Square Error (RMSE). The results for each dataset are presented separately. For the classification task, F-measure or F-score is denoted by $F_{1}$, and is defined as,

(19) $$F_{1}=2\times \left(\frac{precision\;\times recall}{precision+recall}\right)\;$$

The accuracy value is calculated as,

(19) $$Accuracy=\;\frac{TP\;+\;TN}{TP+TN+FP+FN}$$

where TP = true positive, TN = true negative, FP = false positive, FN = false negative. The RMSE value is calculated as,

(21) $$RMSE=\;\sqrt{\frac{1}{N}\overset{N}{\underset{i=1}{\sum }}{\left({\hat{S}}_{i}-S_{i}\right)}^{2}}$$

where ${\hat{S}}_{i}$ and $S_{i}$ represents the predicted signal and observed signal respectively.

### 4.1. HAR Evaluation

**Figure 2:**
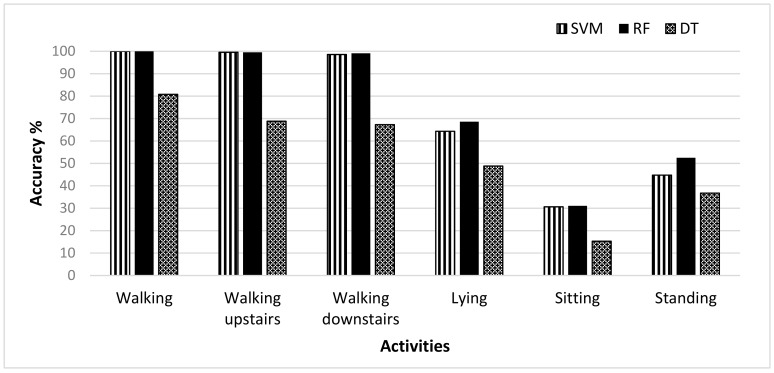
User recognition accuracy obtained for support vector machine (SVM), random forest (RF), and decision tree (DT) classifiers for selected activities in the Human Activity Recognition using Smartphone dataset (HAR) dataset.

**Figure 3:**
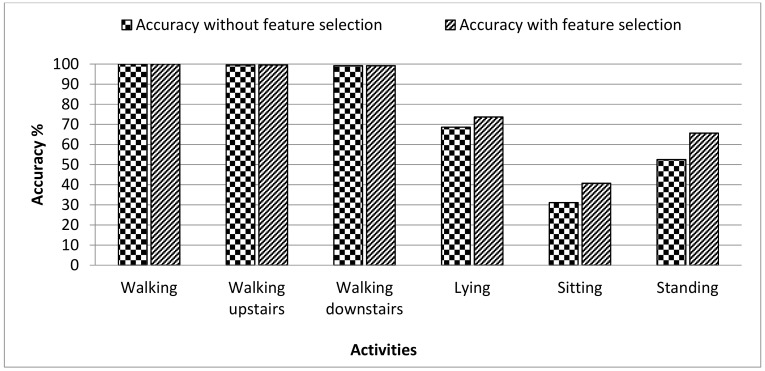
Comparison of user recognition accuracies achieved by RF classifier on the HAR dataset with and without feature selection.

The HAR dataset included the six basic activities, i.e., walking, sitting, standing, lying, walking upstairs, and walking downstairs. Extensive results are presented by implementing three classifiers SVM, RF, and DT. The average accuracy of user recognition was highest in case of the RF classifier which was 75.14%, the average accuracy of the SVM classifier was lower than the RF which was 72.98%, and DT showed the lowest average accuracy of 52.95% among all. Table 2 shows the results of the three classifiers in terms of performance metrics. In the case of SVM and RF classifier, the user recognition accuracy was better for walking, walking upstairs, and walking downstairs activities because of their dynamic nature as the walking pattern of everyone is different. In the case of the walking activity, the SVM classifier showed 100% while the RF classifier showed 99.54% recognition accuracy. However, the RMSE was lower in the case of the RF classifier compared to the SVM classifier. For static activities, sitting and standing, the recognition accuracy was low for all three classifiers and this was due to the reason that the static activities do not involve movement and their still nature make it difficult to recognize the user based on these patterns. Figure 2 shows the recognition accuracy of the three classifiers for six activities in the HAR dataset. The accuracy of these classifiers was calculated without any feature selection method. The RF classifier showed the highest accuracies among other classifiers when no feature selection was applied. A wrapper feature selection method, i.e., WrapperSubsetEval, was also applied on the dataset to reduce the dimensionality of the feature vector by selecting more descriptive features, and the accuracy was calculated by the RF classifier. Figure 3 shows the comparison of accuracies with and without the feature selection method obtained by the RF classifier. It is clear from the figure that the accuracy obtained after the feature selection method was higher for all activities in the dataset. Those activities which already have higher accuracy, were not much influenced by feature selection. However, for static activities, there was an improvement in the results with accuracies of lying 5.04%, sitting 9.66% and standing 13.13%. So, the feature selection method, when applied with the RF classifier, showed improvement in the accuracy of the HAR dataset. Moreover, Table 3 also contains the confusion matrix of 30 users, which shows the results of user identification for the walking activity. Only the best results of user identification are shown, and it can be seen that no data is misclassified.

### 4.2. MobiAct Evaluation

Next, we evaluated performance based on the MobiAct dataset. Out of twelve, nine activities were used in this research study, which was more related to user authentication. For example, walking, sitting, standing, etc. are more common activities in daily life, and by monitoring these type of activities, a user can easily be authenticated. As the number of sensors was larger in the MobiAct dataset than in the HAR dataset, the length of the feature vector was increased. For each data sample, the same features were computed.

The results evaluated on the MobiAct dataset are shown in Table 4. The random forests (average accuracy = 96.03%) classifier shows the best classification results against SVM (average accuracy = 91.31%) and decision tree (average accuracy = 76.28%) because the RF classifier added randomness to the model and thus produced more stable and accurate results. The worst recognition accuracy was achieved by the DT classifier for all activities except walking. The same approach was employed, but this dataset was allowed to evaluate the framework in a complex way due to a large number of activities compared to the other two datasets and the nature of activities. The recognition accuracy wass higher in the case of walking, jogging, and stairs up (dynamic activities). Based on the transitional activity, sit to stand, users were recognized accurately as the recognition accuracy was 100%, meaning that every user was identified correctly, and no user was misclassified or recognized as another individual. In this dataset, the user recognition accuracy on static activities, standing and sitting on the chair, was high and improved compared to the HAR dataset.

**Figure 4:**
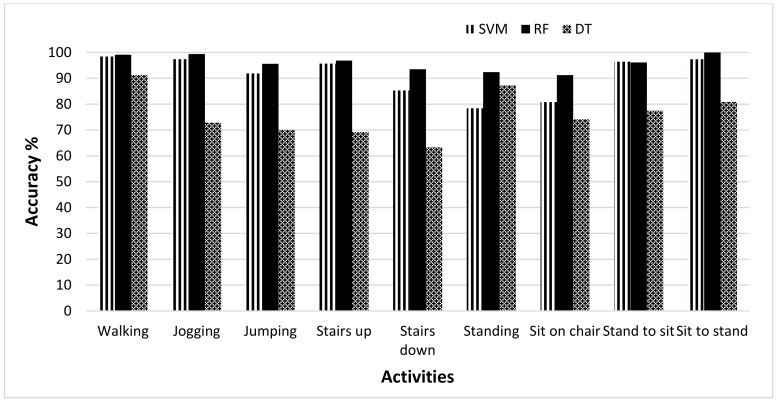
User recognition accuracy of RF, SVM, and DT classifiers for selected activities in MobiAct dataset.

**Figure 5:**
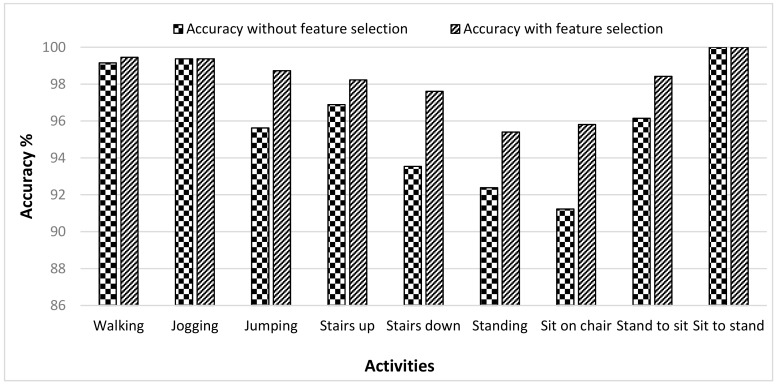
Comparison of recognition accuracies obtained by RF classifier for MobiAct dataset with and without feature selection.

Figure 4 shows the recognition accuracy of three different classifiers based on selected activities when the no feature selection method was applied. It was observed that the best recognition accuracy was achieved by the RF classifier. For the activities; stairs down, standing and sitting on the chair, there was a large gap between the accuracies of SVM and RF. The DT, however, showed the worst performance among all. Its performance was less than the other two classifiers except in the case of the standing activity. In that case, it showed better performance compared to SVM. This showed that the RF classifier performed best in recognizing users from their activity patterns. Figure 5 clearly demonstrates the increase in accuracy of MobiAct dataset achieved through the RF classifier when the feature selection was applied. A comparison was made between the accuracies when calculated using all the features, and when the feature selection method was applied. The activities of jumping, stairs down, standing, and sit-on-chair showed a significant increase in the accuracies of 3.11%, 4.07%, 3.02%, and 4.58% respectively. The other activities, including walking, stairs up and stand-to-sit, showed only a little amount of increase in the accuracy of 0.3% to 2.27%, while sit-to-stand activity showed an accuracy of 100% with both approaches (with and without feature selection).

### 4.3. PAMAP2 Evaluation

The PAMAP2 dataset did not include transitional activities, so the analysis was done on static and dynamic activities. The results of the dataset were calculated at three different body positions (chest, hand, and ankle) and shown in the form of performance metrics in Table 5. By examining the table, it was analyzed that the RF classifier showed the best results for user recognition due to its ability of automatic feature selection and stability property. The average accuracy of the RF (93.44%) classifier was higher compared to SVM (92.39%) and DT (85.02%) due to its automatic feature selection ability and stability.

The classification results also depicted the effect of sensor placement at different body positions. By analyzing the results, the ankle was considered the best position in recognizing the user as it had provided satisfactory results in identifying users for all activities. The highest user recognition accuracy was achieved in the case of Nordic walking (97.8%), running (96.8%), and walking (97.4%) activities. Based on the activities of Nordic walking, walking, running, lying and upstairs, sensor placement at the chest position also showed better results for user identification. As the recognition accuracy was low when the sensors placed at hand position, this position was considered not good enough for discriminating users.

**Figure 6:**
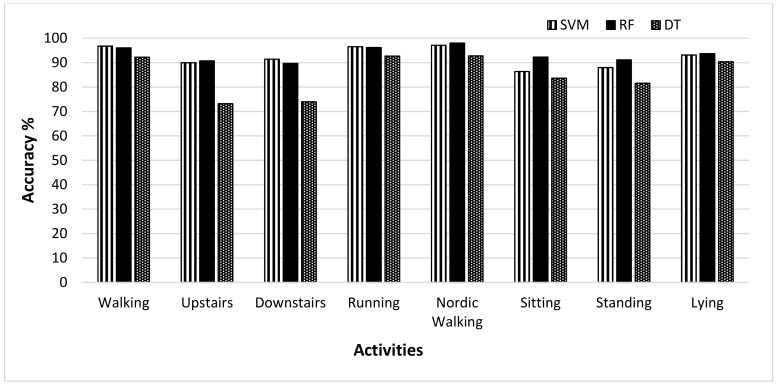
User recognition accuracy of RF, SVM, and DT classifiers for selected activities in the PAMAP2 dataset.

**Figure 7:**
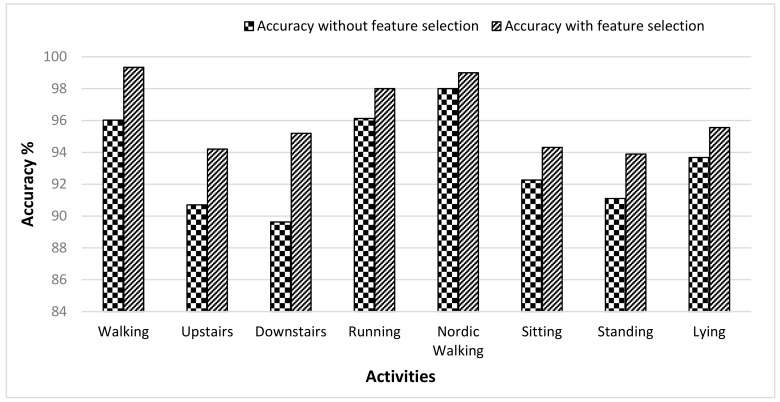
Comparison of user recognition accuracies obtained by the RF classifier for the PAMAP2 dataset with and without feature selection.

Figure 6 shows the average accuracy rate achieved by each classifier for all eight activities in the PAMAP2 dataset. Since for each activity, three different body positions were analyzed, and the average accuracy rate was calculated by taking the mean of recognition accuracies on three represented body positions. It is observed from the graph that the average accuracy rate of the RF classifier was almost equal to the average accuracy rate of the SVM classifier based on walking, running, Nordic walking, lying, and downstairs activities. For the activities sitting and standing, the RF classifier performed better than the SVM. The overall lowest accuracy was achieved by the DT classifier. From the above discussion, it can be concluded that the RF classifier achieved better results than SVM and DT. The accuracy of the RF classifier was further improved by applying a wrapper feature selection method. It is illustrated in Figure 7 that the performance of the RF classifier resulted in higher accuracy when the feature selection method was applied. For the activities of walking, walking upstairs and walking downstairs, the difference between the accuracies (obtained with and without feature selection) was about 3.32%, 3.51%, and 5.57% respectively. Remaining activities also showed improvement in accuracy with a feature selection approach, and the rise in accuracy was from 1% to 2.79%. To provide the individual accuracies for all the users, Table 6 shows the confusion matrix of user recognition for the PAMAP2 dataset. Most of the examples lie on the diagonal except for a few cases.

**Figure 8:**
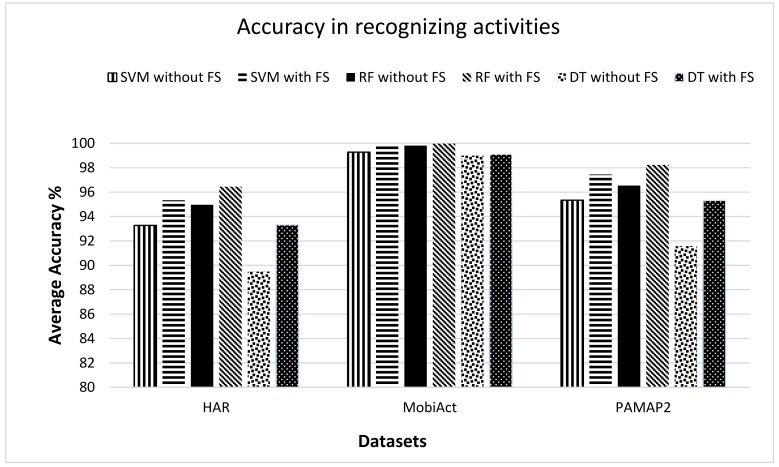
Comparison of average accuracy for the SVM, RF, and DT classifier with and without feature selection (FS) on selected datasets based on activity recognition.

Figure 8 shows the performance of the selected classifier on three different datasets for recognizing activities. The performance of the selected classifier was evaluated in terms of average accuracy. A comparison of selected classifiers was made on the basis of accuracies obtained without and with the feature selection (FS) approach, which shows the accuracy with FS was better compared to the accuracy without FS. Another comparison of evaluated performance was made on three datasets, which showed that the best classification accuracy was always achieved by the RF classifier and the worst accuracy by the DT classifier for activity recognition in case of any dataset. By comparing the results of different datasets, the MobiAct dataset showed the highest average accuracy achieved by all classifiers compared to other datasets. For activity recognition, the average accuracy rate of the RF classifier (with FS) in the case of the MobiAct dataset was 99.98% which was 0.06% and 0.76% higher than the average accuracy rate of the SVM and DT classifiers respectively.

Table 7 shows the comparison of our approach with existing work related to continuous authentication of the user with different datasets and number of activities. Wu et al. and Ehatisham-ul-haq et al. presented an authentication scheme for smartphone using the smartphone’s inertial sensors that achieved an accuracy of 98.5% and 99.18% respectively. Other approaches performing identification achieved an EER of 5.7% [60] and False Acceptance Rate (FAR) and False Rejection Rate (FRR) of 5.01% and 6.85% [61] over a small number of activities. Our proposed approach proved to be better compared to existing work mentioned for the following reasons: (1) It considers multiple datasets of heterogeneous sensors consisting of a smartphone’s built-in inertial sensors as well as on-body wearable sensors. (2) The number of chosen activities appeared to be higher compared to other works, i.e., a total of 12 distinct activities over all datasets. (3) The average accuracy (99.81%) on the MobiAct dataset with the highest number of population, outperformed the results obtained by other works.

## 5. Conclusions and Future Work

In this work, a heterogeneous framework was proposed for implicit and continuous user authentication. The proposed framework was tested on three different datasets, two composed of smartphone motion sensors and one wearable inertial measurement unit (IMU). The sensor’s data of each dataset was preprocessed and then classified one at a time using three prevalent classifiers DT, SVM, and RF. At first, the activities were recognized from sensors data, and then user identification was performed by analyzing the physical activity patterns of the users. The results were calculated in terms of performance metrics accuracy, F-measure, and RMSE. A comparison was made on performance among the classifiers. The results demonstrate that the RF classifier shows the best results among all the classifiers for every dataset. To improve the accuracy, a wrapper feature selection method was applied and results with feature selection were only computed by the RF classifier and compared in terms of accuracy. The best results were obtained on the MobiAct dataset for activity recognition as well as user recognition. The average recognition accuracy of the RF classifier for the MobiAct dataset was 97.13% when the feature selection was employed. Even standing and sit-on-chair have low accuracy compared to accuracies of other activities, but still, the accuracy did not drop below 95%, which indicated the reliability of the suggested solution. The average accuracy of the HAR dataset was 80%, which was quite low compared to the MobiAct dataset, and that was due to the lower accuracy of static activities, i.e., lying, sitting, and standing. This is in contrast with PAMAP2 dataset in which user recognition was performed by placing sensors at different body positions and average user recognition accuracy obtained at hand, chest, and ankle was 91.56%, 95.62%, and 97.84% respectively. This also reveals useful information about the placement of sensors on the body. The ankle position was found suitable for placing sensors for user recognition.

Although the currently implemented solution provides satisfactory results for smartphone and wearable sensors, as future research work, exploring new substitutes of the proposed solution can be beneficial in improving the accuracy. Other possible future work is to further improve user recognition by incorporating a large number of activities such as complex and transitional activities to make a generic framework. Similarly, in this work the datasets consist of smartphone sensors data considered only one position for the smartphone, but in the future, a comprehensive smartphone dataset can be considered which involves multiple body positions for smartphone placements. In this way, position-aware user identification can be performed to improve the recognition results.

## Figures and Tables

**Table 1:** Characteristics of the selected datasets.

Dataset	Sensors	No. of Users	No. of Activities	Activities		
				Static	Dynamic	Transitions
HAR [26]	2 Acc 1 Gyro	30	6	Standing Sitting Lying down	Walking Walking upstairs Walking downstairs	-
PAMAP2 [27]	3 IMUs: 2 Acc 1 Gyro 1 Mag	9	8	Standing Sitting Lying down	Walking Running Nordic Walking Walking upstairs Walking downstairs	-
MobiAct [25]	1 Acc 1 Gyro 1 Mag	59	9	Standing Sitting on chair	Walking Walking upstairs Walking downstairs Jogging Jumping	Sit-to-stand Stand-to-sit

**Table 2:** Results of user recognition based on performance metrics for selected activities in the HAR dataset.

	SVM			RF			DT		
Activities	Accuracy %	F-Measure	RMSE	Accuracy %	F-Measure	RMSE	Accuracy %	F-Measure	RMSE
Walking	100	1.00	0.1743	100	0.996	0.0812	80.82	0.800	0.1153
Walking upstairs	99.54	0.995	0.1743	99.54	0.995	0.0942	68.80	0.683	0.1423
Walking downstairs	98.58	0.985	0.1743	99.13	0.991	0.1089	67.25	0.672	0.1524
Lying	64.34	0.645	0.1749	68.60	0.668	0.1377	48.83	0.476	0.1756
Sitting	30.67	0.291	0.1769	31.09	0.311	0.1704	15.28	0.152	0.2254
Standing	44.78	0.438	0.1754	52.50	0.512	0.1533	36.74	0.369	0.1972

**Table 3:** Confusion matrix of user identification for walking activity in the HAR dataset.

	U 1	U 2	U 3	U 4	U 5	U 6	U 7	U 8	U 9	U 10	U 11	U 12	U 13	U 14	U 15	U 16	U 17	U 18	U 19	U 20	U 21	U 22	U 23	U 24	U 25	U 26	U 27	U 28	U 29	U 30
U1	13	0	0	0	0	0	0	0	0	0	0	0	0	0	0	0	0	0	0	0	0	0	0	0	0	0	0	0	0	0
U2	0	8	0	0	0	0	0	0	0	0	0	0	0	0	0	0	0	0	0	0	0	0	0	0	0	0	0	0	0	0
U3	0	0	8	0	0	0	0	0	0	0	0	0	0	0	0	0	0	0	0	0	0	0	0	0	0	0	0	0	0	0
U4	0	0	0	8	0	0	0	0	0	0	0	0	0	0	0	0	0	0	0	0	0	0	0	0	0	0	0	0	0	0
U5	0	0	0	0	7	0	0	0	0	0	0	0	0	0	0	0	0	0	0	0	0	0	0	0	0	0	0	0	0	0
U6	0	0	0	0	0	8	0	0	0	0	0	0	0	0	0	0	0	0	0	0	0	0	0	0	0	0	0	0	0	0
U7	0	0	0	0	0	0	8	0	0	0	0	0	0	0	0	0	0	0	0	0	0	0	0	0	0	0	0	0	0	0
U8	0	0	0	0	0	0	0	7	0	0	0	0	0	0	0	0	0	0	0	0	0	0	0	0	0	0	0	0	0	0
U9	0	0	0	0	0	0	0	0	7	0	0	0	0	0	0	0	0	0	0	0	0	0	0	0	0	0	0	0	0	0
U10	0	0	0	0	0	0	0	0	0	7	0	0	0	0	0	0	0	0	0	0	0	0	0	0	0	0	0	0	0	0
U11	0	0	0	0	0	0	0	0	0	0	8	0	0	0	0	0	0	0	0	0	0	0	0	0	0	0	0	0	0	0
U12	0	0	0	0	0	0	0	0	0	0	0	7	0	0	0	0	0	0	0	0	0	0	0	0	0	0	0	0	0	0
U13	0	0	0	0	0	0	0	0	0	0	0	0	8	0	0	0	0	0	0	0	0	0	0	0	0	0	0	0	0	0
U14	0	0	0	0	0	0	0	0	0	0	0	0	0	8	0	0	0	0	0	0	0	0	0	0	0	0	0	0	0	0
U15	0	0	0	0	0	0	0	0	0	0	0	0	0	0	7	0	0	0	0	0	0	0	0	0	0	0	0	0	0	0
U16	0	0	0	0	0	0	0	0	0	0	0	0	0	0	0	7	0	0	0	0	0	0	0	0	0	0	0	0	0	0
U17	0	0	0	0	0	0	0	0	0	0	0	0	0	0	0	0	8	0	0	0	0	0	0	0	0	0	0	0	0	0
U18	0	0	0	0	0	0	0	0	0	0	0	0	0	0	0	0	0	7	0	0	0	0	0	0	0	0	0	0	0	0
U19	0	0	0	0	0	0	0	0	0	0	0	0	0	0	0	0	0	0	7	0	0	0	0	0	0	0	0	0	0	0
U20	0	0	0	0	0	0	0	0	0	0	0	0	0	0	0	0	0	0	0	7	0	0	0	0	0	0	0	0	0	0
U21	0	0	0	0	0	0	0	0	0	0	0	0	0	0	0	0	0	0	0	0	7	0	0	0	0	0	0	0	0	0
U22	0	0	0	0	0	0	0	0	0	0	0	0	0	0	0	0	0	0	0	0	0	6	0	0	0	0	0	0	0	0
U23	0	0	0	0	0	0	0	0	0	0	0	0	0	0	0	0	0	0	0	0	0	0	8	0	0	0	0	0	0	0
U24	0	0	0	0	0	0	0	0	0	0	0	0	0	0	0	0	0	0	0	0	0	0	0	8	0	0	0	0	0	0
U25	0	0	0	0	0	0	0	0	0	0	0	0	0	0	0	0	0	0	0	0	0	0	0	0	10	0	0	0	0	0
U26	0	0	0	0	0	0	0	0	0	0	0	0	0	0	0	0	0	0	0	0	0	0	0	0	0	8	0	0	0	0
U27	0	0	0	0	0	0	0	0	0	0	0	0	0	0	0	0	0	0	0	0	0	0	0	0	0	0	8	0	0	0
U28	0	0	0	0	0	0	0	0	0	0	0	0	0	0	0	0	0	0	0	0	0	0	0	0	0	0	0	7	0	0
U29	0	0	0	0	0	0	0	0	0	0	0	0	0	0	0	0	0	0	0	0	0	0	0	0	0	0	0	0	7	0
U30	0	0	0	0	0	0	0	0	0	0	0	0	0	0	0	0	0	0	0	0	0	0	0	0	0	0	0	0	0	9

**Table 4:** Results of user recognition based on performance metrics for selected activities in the MobiAct dataset.

	SVM			RF			DT		
Activities	Accuracy %	F-Measure	RMSE	Accuracy %	F-Measure	RMSE	Accuracy %	F-Measure	RMSE
Walking	98.46	0.985	0.1252	99.15	0.991	0.0453	91.36	0.914	0.0522
Jogging	97.44	0.975	0.1252	99.37	0.993	0.0682	72.80	0.721	0.0949
Jumping	91.89	0.928	0.1252	95.62	0.955	0.0771	70.03	0.700	0.0986
Stairs up	95.71	0.956	0.1252	96.89	0.969	0.0787	69.21	0.694	0.1013
Stairs down	85.31	0.852	0.1253	93.54	0.934	0.0876	63.36	0.620	0.1077
Standing	78.43	0.786	0.1253	92.38	0.926	0.0616	87.32	0.873	0.0599
Sit on chair	80.82	0.812	0.2141	91.23	0.902	0.0589	74.18	0.751	0.1662
Stand to sit	96.42	0.963	0.1252	96.15	0.769	0.0708	77.46	0.778	0.086
Sit to stand	97.36	0.974	0.213	100	1.00	0.1224	80.83	0.803	0.1433

**Table 5:** Results of user recognition based on performance metrics for selected activities in the Physical Activity Monitoring dataset (PAMAP2).

	SVM			RF			DT			
Activities	Accuracy %	F-Measure	RMSE	Accuracy%	F-Measure	RMSE	Accuracy%	F-Measure	RMSE	Body Position
Walking	94.89	0.949	0.293	93.61	0.881	0.1245	89.08	0.938	0.1659	Hand
	97.02	0.970	0.292	97.02	0.970	0.099	92.67	0.927	0.1283	Chest
	98.29	0.983	0.292	97.44	0.975	0.0787	94.89	0.949	0.1129	Ankle
Upstairs	82.30	0.822	0.297	84.30	0.842	0.211	60.17	0.605	0.2985	Hand
	93.80	0.939	0.294	93.90	0.939	0.1659	81.41	0.813	0.211	Chest
	93.80	0.939	0.294	93.90	0.938	0.1777	77.87	0.773	0.2336	Ankle
Downstairs	87.12	0.870	0.295	87.15	0.853	0.207	67.33	0.667	0.2813	Hand
	90.09	0.901	0.293	88.67	0.887	0.19	74.27	0.725	0.2634	Chest
	97.02	0.970	0.292	93.08	0.930	0.1707	80.21	0.749	0.2262	Ankle
Running	95.78	0.958	0.313	95.78	0.958	0.123	93.68	0.937	0.1455	Hand
	95.78	0.958	0.313	95.78	0.958	0.1225	93.68	0.937	0.1455	Chest
	97.89	0.978	0.312	96.84	0.966	0.1192	90.52	0.903	0.1777	Ankle
Nordic walking	96.73	0.984	0.302	97.36	0.968	0.1089	90.21	0.902	0.1611	Hand
	97.28	0.973	0.302	97.82	0.979	0.1063	91.30	0.913	0.1536	Chest
	97.28	0.973	0.302	97.82	0.979	0.084	96.73	0.967	0.0965	Ankle
Sitting	80.66	0.805	0.296	87.84	0.879	0.1845	80.15	0.792	0.2231	Hand
	83.97	0.842	0.295	92.81	0.929	0.151	81.76	0.816	0.2089	Chest
	94.47	0.945	0.293	96.13	0.961	0.1083	88.95	0.890	0.1616	Ankle
Standing	79.56	0.974	0.297	87.48	0.875	0.1829	75.80	0.754	0.2367	Hand
	91.39	0.915	0.293	91.93	0.920	0.1479	83.33	0.836	0.2001	Chest
	93.01	0.930	0.293	93.89	0.936	0.1339	85.55	0.864	0.1795	Ankle
Lying	92.52	0.905	0.294	92.57	0.926	0.1232	87.36	0.873	0.1736	Hand
	93.68	0.937	0.292	94.81	0.947	0.11	92.63	0.926	0.1358	Chest
	93.15	0.932	0.295	93.67	0.937	0.1142	91.05	0.910	0.1447	Ankle

**Table 6:** Confusion matrix of user identification for the Nordic walking activity in PAMAP2 dataset.

	U1	U2	U3	U4	U5	U6	U7	U8
U1	19	0	0	0	0	0	1	19
U2	1	28	0	0	0	0	0	1
U3	0	0	27	0	0	0	0	0
U4	0	0	1	25	0	0	0	0
U5	0	0	1	0	25	0	0	0
U6	0	0	0	1	0	27	0	0
U7	0	0	0	0	0	0	28	0
U8	19	0	0	0	0	0	1	19

**Table 7:** Comparisons of previous work-related continuous authentication with our approach.

Study	Dataset	No. of Users	Sensor Type	No. of Activities	Results
Shen et al. [61]	[62]	48	Smartphone	1 (Passcode)	FAR = 5.01% FRR = 6.85%
Damaševičius et al. [60]	USC-HAD [63]	14	Wearable	1 (Gait recognition)	EER = 5.7%
Wu et al. [64]	[65]	40	Wearable	6	Accuracy = 98.5% F1-score = 86.67%
Ehatisham-ul-haq et al. [41]	[66]	10	Smartphone	6	Avg. accuracy = 99.18%
de Fuentes et al. [67]	Sherlock dataset [68]	50	Smartphone	N/A	Accuracy = 97.05%
Our approach	HAR [26], MobiAct [25], PAMAP2 [27]	30, 59, 9	Smartphone + Wearable	6, 9, 8	Accuracy = 94.96%, 99.81%, 96.54%

## References

[1] Zeng E., Mare S., Roesner F. End user security and privacy concerns with smart homes. Proceedings of the Thirteenth Symposium on Usable Privacy and Security (SOUPS 2017).

[2] Wei J., Hu X., Liu W. (2012). An improved authentication scheme for telecare medicine information systems. J. Med. Syst..

[3] Alzubaidi A., Kalita J. (2016). Authentication of smartphone users using behavioral biometrics. IEEE Commun. Surv. Tutor..

[4] Delac K., Grgic M. A survey of biometric recognition methods. Proceedings of the 46th International Symposium Electronics in Marine.

[5] Kelley P.G., Komanduri S., Mazurek M.L., Shay R., Vidas T., Bauer L., Christin N., Cranor L.F., Lopez J. Guess again (and again and again): Measuring password strength by simulating password-cracking algorithms. Proceedings of the IEEE Symposium on Security and Privacy (SP).

[6] Ma J., Yang W., Luo M., Li N. A study of probabilistic password models. Proceedings of the 2014 IEEE Symposium on Security and Privacy (SP).

[7] Owusu E., Han J., Das S., Perrig A., Zhang J. ACCessory: Password inference using accelerometers on smartphones. Proceedings of the Twelfth Workshop on Mobile Computing Systems & Applications.

[8] Aviv A.J., Gibson K.L., Mossop E., Blaze M., Smith J.M. (2010). Smudge Attacks on Smartphone Touch Screens. Woot.

[9] Schaub F., Deyhle R., Weber M. Password entry usability and shoulder surfing susceptibility on different smartphone platforms. Proceedings of the 11th International Conference on Mobile and Ubiquitous Multimedia.

[10] Mobile Users Can’t Leave Their Phone alone for Six Minutes and Check It up to 150 Times a Day. http://www.dailymail.co.uk/news/article-2276752/Mobileusers-leave-phone-minutes-check-150-times-day.html.

[11] Tamviruzzaman M., Ahamed S.I., Hasan C.S., O’brien C. ePet: When cellular phone learns to recognize its owner. Proceedings of the 2nd ACM workshop on Assurable and usable security configuration.

[12] Jain A.K., Ross A., Prabhakar S. (2004). An introduction to biometric recognition. IEEE Trans. Circuits Syst. Video Technol..

[13] Seneviratne S., Hu Y., Nguyen T., Lan G., Khalifa S., Thilakarathna K., Hassan M., Seneviratne A. (2017). A survey of wearable devices and challenges. IEEE Commun. Surv. Tutor..

[14] Frank M., Biedert R., Ma E., Martinovic I., Song D. (2013). Touchalytics: On the applicability of touchscreen input as a behavioral biometric for continuous authentication. IEEE Trans. Inf. Forensics Secur..

[15] Trojahn M., Ortmeier F. Toward mobile authentication with keystroke dynamics on mobile phones and tablets. Proceedings of the 2013 27th International Conference on Advanced Information Networking and Applications Workshops (WAINA).

[16] Zheng N., Bai K., Huang H., Wang H. You Are How You Touch: User Verification on Smartphones via Tapping Behaviors. Proceedings of the IEEE 22nd International Conference on Network Protocols.

[17] Ferrero R., Gandino F., Montrucchio B., Rebaudengo M., Velasco A., Benkhelifa I. On gait recognition with smartphone accelerometer. Proceedings of the 4th Mediterranean Conference on Embedded Computing (MECO).

[18] Nickel C., Wirtl T., Busch C. Authentication of smartphone users based on the way they walk using k-nn algorithm. Proceedings of the 2012 Eighth International Conference on Intelligent Information Hiding and Multimedia Signal Processing (IIH-MSP).

[19] Buthpitiya S., Zhang Y., Dey A.K., Griss M. (2011). N-gram geo-trace modeling. Proceedings of the International Conference on Pervasive Computing.

[20] Shen C., Chen Y., Guan X. (2018). Performance evaluation of implicit smartphones authentication via sensor-behavior analysis. Inf. Sci..

[21] Ehatisham-ul-Haq M., Azam M.A., Naeem U., Amin Y., Loo J. (2018). Continuous authentication of smartphone users based on activity pattern recognition using passive mobile sensing. J. Netw. Comput. Appl..

[22] Sun L., Zhang D., Li B., Guo B., Li S. (2010). Activity recognition on an accelerometer embedded mobile phone with varying positions and orientations. Proceedings of the International Conference on Ubiquitous Intelligence and Computing.

[23] Chen Y., Shen C. (2017). Performance analysis of smartphone-sensor behavior for human activity recognition. IEEE Access.

[24] Derawi M.O., Nickel C., Bours P., Busch C. Unobtrusive user-authentication on mobile phones using biometric gait recognition. Proceedings of the 2010 Sixth International Conference on Intelligent Information Hiding and Multimedia Signal Processing.

[25] Chatzaki C., Pediaditis M., Vavoulas G., Tsiknakis M. Human daily activity and fall recognition using a smartphone’s acceleration sensor. Proceedings of the International Conference on Information and Communication Technologies for Ageing Well and e-Health.

[26] Anguita D., Ghio A., Oneto L., Parra X., Reyes-Ortiz J.L. A public domain dataset for human activity recognition using smartphones. Proceedings of the European Symposium on Artificial Neural Networks (ESANN).

[27] Reiss A., Stricker D. Introducing a new benchmarked dataset for activity monitoring. Proceedings of the 2012 16th International Symposium on Wearable Computers (ISWC).

[28] Bao L., Intille S.S. Activity recognition from user-annotated acceleration data. Proceedings of the International Conference on Pervasive Computing.

[29] Lee S.-W., Mase K. (2002). Activity and location recognition using wearable sensors. IEEE Pervasive Comput..

[30] Bulling A., Blanke U., Schiele B. (2014). A tutorial on human activity recognition using body-worn inertial sensors. ACM Comput. Surv. (CSUR).

[31] Plötz T., Hammerla N.Y., Olivier P. Feature learning for activity recognition in ubiquitous computing. Proceedings of the International Joint Conference on Artificial Intelligence (IJCAI).

[32] Yang G.-Z., Yang G. (2006). Body Sensor Networks.

[33] Shoaib M. (8–12 September 2013). Human activity recognition using heterogeneous sensors. Proceedings of the 2013 ACM Conference on Ubiquitous Computing.

[34] Gafurov D., Helkala K., Søndrol T. (2006). Biometric Gait Authentication Using Accelerometer Sensor. JCP.

[35] Blasco J., Peris-Lopez P. (2018). On the Feasibility of Low-Cost Wearable Sensors for Multi-Modal Biometric Verification. Sensors.

[36] Zhang Y., Gravina R., Lu H., Villari M., Fortino G. (2018). PEA: Parallel electrocardiogram-based authentication for smart healthcare systems. J. Netw. Comput. Appl..

[37] Li X., Ibrahim M.H., Kumari S., Sangaiah A.K., Gupta V., Choo K.-K.R. (2017). Anonymous mutual authentication and key agreement scheme for wearable sensors in wireless body area networks. Comput. Netw..

[38] Cola G., Avvenuti M., Musso F., Vecchio A. Gait-based authentication using a wrist-worn device. Proceedings of the 13th International Conference on Mobile and Ubiquitous Systems: Computing, Networking and Services.

[39] Xu W., Shen Y., Zhang Y., Bergmann N., Hu W. Gait-watch: A context-aware authentication system for smart watch based on gait recognition. Proceedings of the Second International Conference on Internet-of-Things Design and Implementation.

[40] Yang J., Li Y., Xie M. MotionAuth: Motion-based authentication for wrist worn smart devices. Proceedings of the 2015 IEEE International Conference on Pervasive Computing and Communication Workshops (PerCom Workshops).

[41] Ehatisham-ul-Haq M., Azam M.A., Loo J., Shuang K., Islam S., Naeem U., Amin Y. (2017). Authentication of smartphone users based on activity recognition and mobile sensing. Sensors.

[42] Conti M., Zachia-Zlatea I., Crispo B. Mind how you answer me: Transparently authenticating the user of a smartphone when answering or placing a call. Proceedings of the 6th ACM Symposium on Information, Computer and Communications Security.

[43] Muaaz M., Mayrhofer R. (2017). Smartphone-based gait recognition: From authentication to imitation. IEEE Trans. Mob. Comput..

[44] Abate A.F., Nappi M., Ricciardi S. (2017). I-am: Implicitly authenticate me person authentication on mobile devices through ear shape and arm gesture. IEEE Trans. Syst. Man Cybern. Syst..

[45] Kwapisz J.R., Weiss G.M., Moore S.A. Cell phone-based biometric identification. Proceedings of the 2010 Fourth IEEE International Conference on Biometrics: Theory Applications and Systems (BTAS).

[46] Lee W.-H., Lee R.B. Multi-sensor authentication to improve smartphone security. Proceedings of the 2015 International Conference on Information Systems Security and Privacy (ICISSP).

[47] Primo A., Phoha V.V., Kumar R., Serwadda A. Context-aware active authentication using smartphone accelerometer measurements. Proceedings of the IEEE Conference on Computer Vision and Pattern Recognition Workshops.

[48] The MobiFall and MobiAct Datasets. https://bmi.teicrete.gr/en/the-mobifall-and-mobiact-datasets-2/.

[49] UCI PAMAP2 Physical Activity Monitoring Data Set. http://archive.ics.uci.edu/ml/datasets/pamap2+physical+activity+monitoring.

[50] Liu R., Zhou J., Liu M., Hou X. A wearable acceleration sensor system for gait recognition. Proceedings of the 2007 2nd IEEE Conference on Industrial Electronics and Applications.

[51] Mostayed A., Kim S., Mazumder M.M.G., Park S.J. Foot step based person identification using histogram similarity and wavelet decomposition. Proceedings of the 2008 International Conference on Information Security and Assurance (ISA 2008).

[52] Reyes-Ortiz J.-L., Oneto L., Samà A., Parra X., Anguita D. (2016). Transition-aware human activity recognition using smartphones. Neurocomputing.

[53] Kohavi R., John G.H. (1997). Wrappers for feature subset selection. Artif. Intell..

[54] Cortes C., Vapnik V. (1995). Support-vector networks. Mach. Learn..

[55] Kohavi R. Scaling up the accuracy of naive-bayes classifiers: A decision-tree hybrid. Proceedings of the Second International Conference on Knowledge Discovery and Data Mining (KDD).

[56] Su X., Tong H., Ji P. (2014). Activity recognition with smartphone sensors. Tsinghua Sci. Technol..

[57] Breiman L. (2001). Random forests. Mach. Learn..

[58] Platt J., Scholkopf B., Burges C., Smola A. (1998). Fast training of support vector machines using sequential minimal optimization. Advances in Kernel Methods-Support Vector Learning.

[59] Salzberg S.L. (1994). C4.5: Programs for Machine Learning by J. Ross Quinlan. Morgan Kaufmann Publishers, Inc., 1993. Mach. Learn..

[60] Damaševičius R., Maskeliūnas R., Venčkauskas A., Woźniak M. (2016). Smartphone user identity verification using gait characteristics. Symmetry.

[61] Shen C., Yu T., Yuan S., Li Y., Guan X. (2016). Performance analysis of motion-sensor behavior for user authentication on smartphones. Sensors.

[62] Shen C., Yu T.W., Yuan S., Li Y.P., Guan X.H. Motion-Sensor Data for Smartphone Authentication. http://nskeylab.xjtu.edu.cn/people/cshen/?p=327.

[63] Zhang M., SAWCHUK A. A Daily Activity Dataset for Ubiquitous Activity Recognition Using Wearable Sensors. Proceedings of the 2012 ACM Conference on Ubiquitous Computing.

[64] Wu G., Wang J., Zhang Y., Jiang S. (2018). A Continuous Identity Authentication Scheme Based on Physiological and Behavioral Characteristics. Sensors.

[65] Nan W.G., Jian W., Rong Z.Y., Shuai J. Sensor Data for Identity Recognition. http://pan.baidu.com/s/1dE9Shwd.

[66] Shoaib M., Bosch S., Incel O., Scholten H., Havinga P. (2014). Fusion of smartphone motion sensors for physical activity recognition. Sensors.

[67] De Fuentes J., Gonzalez-Manzano L., Ribagorda A. (2018). Secure and Usable User-in-a-Context Continuous Authentication in Smartphones Leveraging Non-Assisted Sensors. Sensors.

[68] Mirsky Y., Shabtai A., Rokach L., Shapira B., Elovici Y. Sherlock vs moriarty: A smartphone dataset for cybersecurity research. Proceedings of the 2016 ACM Workshop on Artificial Intelligence and Security.

